# Associations of Depressive Symptoms and Cognition in the FINGER Trial: A Secondary Analysis of a Randomised Clinical Trial

**DOI:** 10.3390/jcm11051449

**Published:** 2022-03-07

**Authors:** Elisa Neuvonen, Jenni Lehtisalo, Tiia Ngandu, Esko Levälahti, Riitta Antikainen, Tuomo Hänninen, Tiina Laatikainen, Jaana Lindström, Teemu Paajanen, Hilkka Soininen, Timo Strandberg, Jaakko Tuomilehto, Miia Kivipelto, Alina Solomon

**Affiliations:** 1 Institute of Clinical Medicine/Neurology, University of Eastern Finland, 70211 Kuopio, Finland; jenni.lehtisalo@thl.fi (J.L.); hilkka.soininen@uef.fi (H.S.); miia.kivipelto@ki.se (M.K.); alina.solomon@uef.fi (A.S.); 2 Population Health Unit, Finnish Institute for Health and Welfare, 00271 Helsinki, Finland; tiia.ngandu@thl.fi (T.N.); esko.levalahti@thl.fi (E.L.); tiina.laatikainen@thl.fi (T.L.); jaana.lindstrom@thl.fi (J.L.); jaakko.tuomilehto@thl.fi (J.T.); 3 Division of Clinical Geriatrics, Department of Neurobiology, Care Sciences, and Society (NVS), Karolinska Institutet, 171 77 Stockholm, Sweden; 4 Center for Life Course Health Research, University of Oulu, 90014 Oulu, Finland; riitta.antikainen@oulu.fi (R.A.); timo.strandberg@oulu.fi (T.S.); 5 Medical Research Center Oulu, Oulu University Hospital, 90029 Oulu, Finland; 6 Neurocenter, Department of Neurology, Kuopio University Hospital, 70029 Kuopio, Finland; tuomo.hanninen@kuh.fi; 7 Institute of Public Health and Clinical Nutrition, University of Eastern Finland, 70211 Kuopio, Finland; 8 Joint Municipal Authority for North Karelia Social and Health Services (Siun Sote), 80210 Joensuu, Finland; 9 Finnish Institute of Occupational Health, 00032 Helsinki, Finland; teemu.paajanen@ttl.fi; 10 Department of Medicine, University of Helsinki and Helsinki University Hospital, 00029 Helsinki, Finland; 11 Department of Public Health, University of Helsinki, 00014 Helsinki, Finland; 12 National School of Public Health, 28029 Madrid, Spain; 13 South Ostrobothnia Central Hospital, 60220 Seinäjoki, Finland; 14 Diabetes Research Group, King Abdulaziz University, Jeddah 21589, Saudi Arabia; 15 Ageing Epidemiology Research Unit, School of Public Health, Imperial College London, London W6 8RP, UK; 16 Center of Theme Aging, Karolinska University Hospital, 171 64 Stockholm, Sweden

**Keywords:** clinical trial, cognition, dementia, depressive symptoms, prevention

## Abstract

Depression and cognition are associated, but the role of depressive symptoms in lifestyle interventions to prevent dementia needs further study. We investigated the intervention effect on depressive symptoms and their associations with cognition in the Finnish Geriatric Intervention Study to Prevent Cognitive Impairment and Disability (FINGER; NCT01041989), a two-year multidomain lifestyle trial. One thousand two-hundred and sixty individuals (60–77 years) at risk for dementia were randomised into a multidomain intervention (diet, exercise, cognitive training, and vascular/metabolic risk monitoring) or control group (regular health advice). Depressive symptoms (Zung scale) and cognition (modified Neuropsychological Test Battery) were evaluated at baseline, 12, and 24 months. One thousand one-hundred and twenty-five participants had baseline Zung data. Mean Zung score decreased 0.73 (SD 5.6) points in the intervention and 0.36 (5.6) points in the control group, with nonsignificant between-group difference (group × time coefficient −0.006, 95% CI −0.019 to 0.007). Overall, higher baseline Zung score was associated with less improvement in global cognition (−0.140, *p* = 0.005) and memory (−0.231, *p* = 0.005). Participants with clinically significant baseline depressive symptoms (Zung ≥ 40 points) had less intervention benefit to executive functioning (group × time × Zung −0.096, 95% CI −0.163 to −0.028). Change in Zung score was not associated with change in cognition. Clinically significant depressive symptoms warrant more attention when designing dementia-prevention interventions.

## 1. Introduction

Cognitive impairment and dementia are major public health problems. Growing knowledge on modifiable risk factors has emphasised the importance of prevention [1]. The first World Health Organization (WHO) guidelines for risk reduction of cognitive decline and dementia mention, for instance, physical activity, smoking cessation, healthy diet, cognitive and social activities, and management of cardiovascular risk factors and depression [2]. Depression is an important risk factor for dementia [1], and depressive symptoms have been associated with poorer cognition in older age [3]. The link between late-life depression and cognition is complex, i.e., depressive symptoms may also represent prodromal symptoms of dementia, especially regarding vascular dementia [4,5].

Depression is closely connected with lifestyle factors. A Cochrane meta-analysis of randomised controlled trials (RCTs) in people with depression showed exercise to be moderately more effective than a control intervention for reducing depressive symptoms [6]. Physical activity was also protective against depression in a systematic review of observational studies [7]. A meta-analysis of observational studies showed that a healthy dietary pattern was associated with reduced risk of depression [8], and a Mediterranean diet with nuts intervention also reduced depression risk among type 2 diabetics [9]. Bene-ficial effects of lifestyle interventions on mood have additionally been observed in people with, e.g., chronic obstructive pulmonary disease, obesity, and psychotic disorders [10,11,12,13]. Regarding other interventions, an RCT among healthy older adults found that computerised cognitive training improved cognition but had no effect on depressive symptoms [14]. Most studies have investigated lifestyle factors separately, but fewer have considered more complex, multidomain lifestyle interventions. One multidomain trial targeting frailty reported that an intervention including physical activity, dietary supplementation, and cognitive training improved depressive symptoms [15].

In addition to being affected by lifestyle factors, depressive symptoms may impact an individual’s ability to adhere to healthy lifestyle changes and improve their cardiovascular and metabolic risk factors [16,17,18]. They may not necessarily hinder lifestyle imp-rovement, as shown for, e.g., cardiometabolic risk in people at risk for diabetes [19] or physical activity among sedentary people [20], but the role of depressive symptoms in multidomain lifestyle interventions aiming to preventing dementia has so far not been investigated.

Due to the multifactorial nature of dementia and disappointing results from many single-domain interventions for dementia risk reduction, the newest generation of prevention RCTs has been testing multidomain lifestyle interventions targeting multiple risk factors simultaneously [21]. Although no significant intervention benefits on either the primary cognitive outcome or depressive symptoms were reported in the Multidomain Alzheimer Preventive Trial (MAPT) and Prevention of Dementia by Intensive Vascular care (preDIVA) trials [22,23], the Finnish Geriatric Intervention Study to Prevent Cognitive Impairment and Disability (FINGER) trial showed that multidomain lifestyle intervention was beneficial for cognition compared with regular health advice among older persons at risk for dementia [24]. It is important to study the effects of depressive symptoms in the context of dementia-prevention trials to see if they influence the intervention effects on cognition. In this study based on the FINGER trial, we investigated: (i) intervention effects on change in depressive symptoms (secondary outcome), (ii) whether depressive symptoms at baseline modified the previously reported intervention benefits of cognition (post hoc analysis), and (iii) whether the change in depressive symptoms were related to change in cognition (post hoc analysis).

## 2. Materials and Methods

### 2.1. The FINGER Trial Design and Participants

The FINGER trial protocol, recruitment process, and primary findings have been previously published [24,25,26]. In brief, FINGER was a two-year RCT conducted in six centers in Finland. Participants at risk for dementia were recruited from previous population-based surveys [27,28]. Eligibility criteria were age 60–77 years, Cardiovascular Risk Factors, Aging, and Dementia (CAIDE) Dementia Risk Score [29] ≥6 points (range 0–15 points), and cognition at a mean level or slightly lower than expected for age (Consortium to Establish a Registry for Alzheimer’s Disease (CERAD) Word List memory learning score <20 points or Word List recall ≤75%, or Mini-Mental State Examination (MMSE) score ≤26 points) [30,31]. Exclusion criteria were diagnosed or suspected dementia, MMSE <20 points, conditions preventing cooperation or safe engagement in the intervention (based on interview with the study doctor, e.g., major depression; malignant tumor; symptomatic cardiovascular disease; and severe vision, hearing, or communicative impairment), and participation in another trial simultaneously.

Of the 2654 individuals screened for eligibility from 7 September 2009 to 24 November 2011, 1260 were randomised in a 1:1 ratio into a multidomain intervention or control group (computer-generated randomisation was done in blocks of four individuals at each site). Outcome assessors were blinded to group allocation and were not involved in intervention activities. The primary outcome was a change in global cognition (extended and modified version of the Neuropsychological Test Battery, mNTB) [32]. Secondary outcomes included cognitive domains (executive functioning, memory, and processing speed) and depressive symptoms.

The authors assert that all procedures contributing to this work comply with the et-hical standards of the relevant national and institutional committees on human experimentation and with the Helsinki Declaration of 1975, as revised in 2008. All procedures involving human subjects were approved by the Coordinating Ethics Committee of the Hospital District of Helsinki and Uusimaa (approval number 94/13/03/00/2009). Written informed consent was obtained from all subjects. The FINGER trial is registered with ClinicalTrials.gov (NCT01041989).

### 2.2. Intervention

The control group received regular health advice. All participants (control and intervention group) met the study nurse at screening, baseline, 6, 12, and 24 months (blood pressure, weight and BMI, and hip and waist circumference measurements), and the study physician at screening and 24 months (medical history and physical examination). At baseline, the study nurse gave all participants oral and written information and advice on healthy diet and physical, cognitive, and social activities beneficial for management of vascular risk factors and disability prevention. Blood samples were collected at baseline, 6, 12, and 24 months, and laboratory test results were mailed to all participants, with general written information about the clinical significance of measurements, and advice to contact primary health care if needed. The intervention group additionally received a multidomain intervention including all four intervention components, as described earlier [25]. Dietary intervention was based on the Finnish nutrition recommendations and comprised individual counselling (three sessions) and group meetings (6–9 sessions), led by study nutritionists. Physical exercise intervention comprised progressive muscle strength training (1–3 times/week), supervised by the study physiotherapists; independent aerobic exercise (2–5 times/week); and postural balance exercises. Cognitive training included 10 group sessions led by the study psychologists and individually tailored computer-based training sessions (2–3 times/week). Management of vascular and metabolic risk factors was based on national guidelines. The intervention group had additional meetings with the study nurse (at 3, 9, and 18 months) for motivational discussion and assessment of anthropometrics, and with the study physician (at 3, 6, and 12 months) for discussing their individual risk profile, including laboratory results and receiving personalised advice on vascular and metabolic factors. Contact with the participant’s own physician was recommended if a need for drug treatment or adjustment was identified. Intervention duration was two years, and the intervention was completed in February 2014.

### 2.3. Assessment of Depressive Symptoms

**Figure 1:**
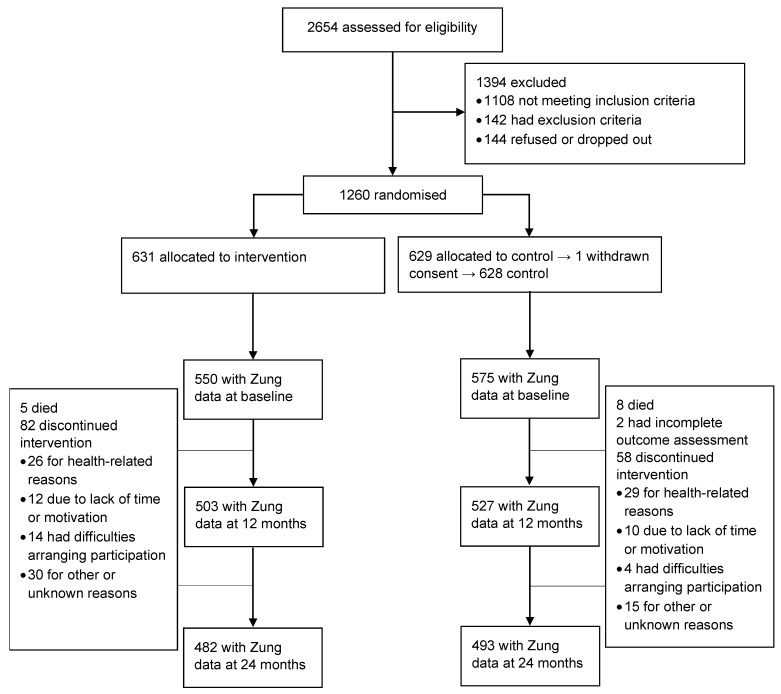
CONSORT flow chart of the study design.

Depressive symptoms were evaluated at baseline, 12-month, and 24-month visits with the Zung Self-Rating Depression Scale (SDS), a 20-item questionnaire capturing the affective, psychological, and somatic symptoms associated with depression [33]. The scale has shown good validity and reliability [34,35]. Half of the items are phrased positively and half negatively, with items scored on a Likert scale ranging from 1 to 4. A total raw score ranging from 20 to 80 can be calculated, with a higher score indicating more severe depressive symptoms. An SDS index can be further calculated by dividing the total raw score by a maximum of 80. These two scores (raw and index) have led to some debate concerning cut-offs for clinically significant depressive symptoms [36]. Originally, a cut-off of 50 for the SDS index (representing a raw score of 40 points) was suggested [37]. In this study, we used this originally suggested cut-off of 40 points in the raw score. A CONSORT flow chart focusing on SDS data is provided as Figure 1.

### 2.4. Assessment of Cognition

Cognition was assessed for global cognition and cognitive domains with mNTB at baseline, 12-month, and 24-month visits [24]. A composite score for global cognition was calculated averaging 14 different tests standardised to z-scores using the total FINGER sample baseline mean and SD, with a higher score indicating better cognitive performance. As secondary cognitive outcomes, mNTB domain z-scores were calculated for executive functioning, memory, and processing speed, as previously described [24,25].

### 2.5. Other Factors

Participants’ annual study visits included anthropometric and blood pressure mea-surements; blood sampling; and questionnaires about sociodemographic factors, lifestyle, quality of life, and general health [24,25]. Self-reported antidepressant medication use was checked with the study doctor at screening and 24-month visit. Based on these data, we created a composite variable of antidepressant use at any timepoint vs. no antidepressant use. To account for the links between depressive symptoms and lifestyle, we calculated a healthy lifestyle change composite index for all participants, based on measures of diet, exercise frequency, and cardiovascular factors (based on the variables used in the FINRISK CVD risk equations) [38], as described earlier [39]. The index was calculated as the mean z-score change (with higher values indicating healthier change) if data were available on at least two of the three components.

### 2.6. Statistical Analyses

We compared baseline characteristics between the intervention and control groups, and between people with and without baseline Zung score data using the χ^2^ test, *t*-test, or Mann–Whitney U test (rank-sum), as appropriate. Mixed-effects regression models with maximum likelihood estimation were used to analyze intervention effects on depressive symptoms (zero-skewness log-transformed Zung score) as a function of the randomisation group, time, and group × time interaction (unadjusted model). Analyses were further adjusted for age, sex, education, trial site, antidepressant use, and healthy lifestyle change index.

We applied a parallel latent growth curve model using a covariance structure analysis framework for examining the associations between Zung score (zero-skewness log-transformed) and cognition. The baseline level (latent intercept) and the change (latent slope) of both Zung score and cognition over the 2-year trial were estimated, and their associations were studied using structural equation modelling (SEM). Maximum likelihood estimation on all available data was used. Graphical presentation of the overall model is shown in Figure S1 in Supplementary material. Analyses were executed grouped based on intervention allocation and initiated with a theoretical full path model (model 0) where all parameters were estimated as unequal between intervention and control groups, as if there were two separate models. Parameters were constrained as equal one at a time, and each model was tested against the full model using the likelihood ratio test (details in Supplementary Methods in Supplementary material). When all constraints were allowed, the model treated the groups as equal (model 5). In more advanced models, some paths were further constrained to [0], and these were shown to be the best-fitting models for all cognitive variables (Supplementary Methods and Table S2 in Supplementary material). We present the results for the best-fitting model and for an intermediate model (model 2), to describe the relations between depressive symptoms and cognition also separately for the intervention and control groups. All SEM analyses were adjusted for age, sex, education, trial site, antidepressant use, and healthy lifestyle change index and were conducted using SPSS Amos (IBM, Chicago, IL, USA), version 25 [40].

We investigated the potential impact of clinically relevant depressive symptoms at baseline (Zung score ≥40 vs. <40 points) on the intervention effects on cognition, using mixed-effects regression model with the best model fit approach. Analyses were initiated with a theoretical full path model, including group × time, group × Zung, time × Zung, and group × time × Zung interactions. The best-fitting models were selected constraining parameters as equal between groups, and each model was compared with the full model using the likelihood ratio test. Bayesian information criterion (BIC) was used to select the best-fitting model. Analyses were adjusted for age, sex, education, trial site, antidepressant use, and healthy lifestyle change index, and were conducted using Stata software for Windows, version 14 (StataCorp, College Station, TX, USA).

## 3. Results

Baseline depressive symptom data were available for 1125 (89.3%) participants. Participants with missing Zung score data included more women and individuals living alone, had a higher body mass index, consumed alcohol less often and fish more often, and had poorer executive functioning compared with participants with available data (Table 1). Baseline demographic, vascular and lifestyle factors, medical history, and cognitive performance were not significantly different between the intervention and control groups (Table S1 in Supplementary material).

### 3.1. Intervention Effects on Change in Zung Score

There was no significant difference in Zung score change between the intervention and control groups (group × time interaction coefficient −0.005, 95% CI −0.018 to 0.007, *p* = 0.40). The result was similar after adjusting for age, sex, education, trial site, antidepressant use, and healthy lifestyle change index (coefficient −0.006, 95% CI −0.019 to 0.007, *p* = 0.35). The observed mean Zung score decreased 0.73 (SD 5.6) points in the intervention and 0.36 (SD 5.6) points in the control group.

### 3.2. Associations between Zung Score and Cognition

Associations of Zung score and cognition are presented in Table 2. Baseline Zung score was inversely associated with baseline cognition, i.e., more pronounced depressive symptoms were related to poorer global cognition (path coefficient −0.302, *p* < 0.001), exe-cutive functioning (−0.360, *p* < 0.001), processing speed (−0.540, *p* < 0.001), and memory (−0.177, *p* = 0.043).

In the intervention and control groups combined, a higher baseline Zung score was associated with less improvement in global cognition (path coefficient −0.140, *p* = 0.005) and memory (−0.231, *p* = 0.005) over two years (Table 2). No associations were found for the intervention or control groups separately, or with changes in other cognitive domains (Table 2).

Change in Zung score was not significantly associated with changes in global cognition or cognitive domains in either allocation group. A decrease in Zung score showed a trend for association with an increase in executive functioning in the control group (Table 2, path coefficient −1.371, *p* = 0.063).

Table 3 (also Table S3 in Supplementary material) shows associations between the dichotomous baseline Zung score (≥40 points, N = 243 vs. <40 points, N = 882 participants) and cognitive change. The full model fit best for executive functioning, whereas the least loaded models with only one interaction term (time × Zung) fit best for global cognition, memory, and processing speed (detailed in Table S4 in Supplementary material). The randomisation group × time × Zung interaction coefficient was −0.096 (95% CI −0.163 to −0.028, *p* = 0.005) for executive functioning, indicating less intervention benefit in this cognitive domain among participants with versus without clinically significant depressive symptoms. Among all participants irrespective of group allocation, Zung score ≥40 points were associated with less improvement in global cognition (time × Zung coefficient −0.035, 95% CI −0.062 to −0.008, *p* = 0.010) and memory (time × Zung coefficient −0.044, 95% CI −0.088 to −0.0001, *p* = 0.049) (Table 3).

## 4. Discussion

In the FINGER multidomain lifestyle RCT, there was no difference in change in dep-ressive symptoms between the intervention and control groups. Overall, more pronounced depressive symptoms at baseline were associated with poorer baseline cognition (global and all domains) and less improvement in global cognition and memory over time, irrespective of intervention allocation. The participants with a baseline Zung score of ≥40 points (suggesting clinically relevant depressive symptoms) seemed to have smaller intervention-related benefits on executive functioning, compared with participants with a Zung score of <40 points.

Although the FINGER intervention had significant benefits on cognition in older adults at risk for dementia from the general population [24], the lack of effect on depressive symptoms was similar to the multidomain MAPT and preDIVA trials [22,23]. Due to its primary focus on early prevention of cognitive decline in at-risk individuals without substantial cognitive impairment, the FINGER trial excluded people with major depression. The mean baseline Zung score was relatively low (33.9 points), leaving less room for improvement compared with RCTs targeting people with depression. The focus on this specific target population, and the exclusion criteria, has probably contributed to the nonsignificant findings regarding intervention effects on depressive symptoms. In addition, in this study, people with missing Zung data included, e.g., more women, individuals living alone, and those with a higher BMI. These factors are also often associated with depressive symptoms, as well as an increased dementia risk [1,4]. Thus, the missing data may have led to an underestimation of the intervention effects on depressive symptoms and their impact on cognition. Previous studies have reported lifestyle-based interventions to be effective in reducing depressive symptoms [6,8,10,12,15]. However, these studies were not primarily designed for preventing cognitive decline or dementia and had different target populations and intervention designs compared with the FINGER.

Depressive symptoms have been associated with poorer cognition in several domains [3]. In line with this, we found that more pronounced depressive symptoms at baseline were related to less cognitive improvement over two years in the FINGER participants irrespective of group allocation. Given the small overall change in depressive symptoms during the trial, it is perhaps not surprising that this was not associated with a change in cognition.

Previous studies have shown that depressive symptoms do not necessarily hinder participants from benefitting from lifestyle interventions [19,20]. However, such studies focused on physical fitness and cardiovascular and diabetes outcomes [19,20], without considering cognition. Our finding, suggesting that clinically relevant depressive symptoms may affect intervention-related benefits to cognition, is particularly interesting in this context. This effect was observed for executive functioning but not global cognition or other cognitive domains. Executive functioning is often the first cognitive domain affected in preclinical Alzheimer’s disease [41] and is also commonly affected in depression [42]. Executive dysfunction is also common in vascular cognitive impairment [43]. There is a well-established association between depression and vascular dementia, although the direction of this association is uncertain [4,43]. Depressive symptomatology may decrease cognitive reserve [44] or may be a prodromal manifestation of dementia-related diseases [4,5], i.e., people with clinically significant depressive symptoms may be closer to cognitive disorder onset and may thus have less room for prevention. Furthermore, it was previously reported that depressive symptoms may affect adherence, dropout, and willingness to participate in intervention studies [16,18]. In the FINGER trial, more pronounced depressive symptoms were related to lower adherence to the physical exercise intervention [45].

The effect of baseline depressive symptoms on the intervention-related benefit to executive functioning was not observed for the continuous Zung score. This may suggest a “threshold effect” related to clinical significance. While the proposed clinical cut-off for the Zung scale has been debated [36], a cut-off of 39–40 is not uncommon in studies with older participants [46,47]. In this study, only 243 participants (21.6%) had a baseline Zung score of ≥40 points. Although we cannot fully confirm the clinical significance (or duration) of their depressive symptoms, our finding suggests that such individuals may require a different approach to prevent cognitive decline, e.g., more tailored support for adherence to a healthy lifestyle, or closer monitoring and early treatment of depression. Our analyses were adjusted for both healthy lifestyle changes (diet, exercise, and cardiovascular factors) and antidepressant medications, but the potential contribution of other lifestyle factors and medication-related factors (drug type, dose, and duration of treatment) could not be fully accounted for. It is also unclear if the observed effect is due to the depressive symptoms themselves or to an underlying dementia-related disease causing both depressive symptoms and poorer cognition. In the latter case, disease-modifying drugs may be needed, in addition to healthy lifestyle changes for preventing/delaying cognitive impairment.

The main strengths of this study include the large, longer-term population-based RCT with a thoroughly designed and conducted multidomain lifestyle intervention and comprehensive repeated measures of both cognition and depressive symptoms [24,25]. The Zung scale has been evaluated in community-based older populations and shown to have acceptable sensitivity and specificity [47].

The study has several limitations. Due to the primary focus of the trial, individuals with major depression were excluded, leaving less room to observe changes in depressive symptoms over time. Although the FINGER population is representative of the at-risk segment of the older Finnish general population without substantial cognitive impairment/dementia [26], as is common in RCTs, recruited individuals may have been more health-conscious and willing to adhere to the intervention than nonparticipants. Participants with missing data on the Zung scale had poorer baseline executive functioning, which may have diluted our findings. Due to the early prevention approach, dementia was not a feasible outcome after two years.

Analyses of the associations between depressive symptoms and cognition were post hoc, and the trial was not powered for three-way interactions. Our findings would thus need to be verified in other multidomain dementia prevention trials [48]. Future studies should also consider the potential impact of neuropathology (e.g., amyloid accumulation) on the interactions between depressive symptoms and cognition in the interventional context [49,50].

## 5. Conclusions

Depressive symptoms affect cognitive performance. If such symptoms are clinically significant, they may also influence the cognitive benefit from lifestyle interventions for dementia risk reduction. Although this finding requires further verification, future dementia-prevention trials may benefit from adapting specific intervention strategies for participants with clinically significant depressive symptoms.

## Figures and Tables

**Table 1:** Comparison of baseline characteristics between people with and without Zung data at baseline.

	*n* (Available /Missing)	Zung Data Available	Zung Data Missing	*p*-Value ^1^
**Demographic characteristics**				
Age (years)	1125/134	69.2 (4.7)	69.9 (4.9)	0.13
Sex: number of women	1125/134	502 (45%)	85 (63%)	**<0.001**
Education (years)	1124/133	10.0 (3.5)	9.4 (3.1)	0.14
Married/cohabiting	1125/133	848 (75%)	86 (65%)	**0.008**
**Vascular factors**				
Systolic blood pressure (mmHg)	1117/133	140 (16.4)	141 (14.5)	0.51
Diastolic blood pressure (mmHg)	1117/133	80 (9.5)	81 (8.9)	0.52
Serum total cholesterol (mmol/L)	1120/134	5.1 (1.0)	5.2 (1.0)	0.29
Fasting plasma glucose (mmol/L)	1122/134	6.1 (0.9)	6.0 (1.0)	0.23
2 h oral glucose tolerance test (mmol/L)	972/112	7.0 (2.2)	7.2 (2.3)	0.39
Body-Mass Index (kg/m^2^)	1115/133	28.1 (4.7)	29.1 (4.5)	**0.010**
Waist circumference (cm)	1116/133	98.2 (12.5)	99.1 (12.1)	0.40
**Lifestyle factors**				
Physically active ≥2/week	1117/129	793 (71%)	89 (69%)	0.64
Current smokers	1123/131	104 (9%)	10 (8%)	0.54
Alcohol drinking ≥1/week	1120/131	515 (46%)	41 (31%)	**0.001**
Fish intake ≥2/week	1122/130	576 (51%)	79 (61%)	**0.042**
Daily vegetable intake	1124/132	698 (62%)	77 (58%)	0.40
**Self-reported medical conditions**				
Hypertension	1118/133	733 (66%)	98 (74%)	0.061
Hypercholesterolemia	1115/134	749 (67%)	90 (67%)	1.00
Diabetes	1119/134	147 (13%)	21 (16%)	0.42
History of myocardial infarction	1119/134	60 (5%)	4 (3%)	0.24
History of stroke	1116/134	59 (5%)	9 (7%)	0.49
Antidepressant use	1125/134	62 (6%)	12 (9%)	0.11
**Cognition**				
Global cognition (mNTB)	1124/134	0.003 (0.57)	−0.08 (0.60)	0.099
Executive functioning (mNTB)	1123/134	0.002 (0.68)	−0.15 (0.66)	**0.017**
Memory (mNTB)	1124/134	0.0005 (0.67)	−0.02 (0.71)	0.52
Processing speed (mNTB)	1124/134	0.01 (0.81)	−0.11 (0.89)	0.17
Mini Mental State Examination	1122/134	26.8 (2.0)	26.6 (2.0)	0.48

Note: Data are presented as mean (SD) for continuous variables and as *n* (%) for categorical variables. mNTB, modified Neuropsychological Test Battery. ^1^ *p*-values < 0.05 are in bold.

**Table 2:** Associations between cognition and the Zung depression score during the FINGER trial.

Path	Path Coefficient (Standard Error)	*p*-Value
**Global cognition**		
Baseline Zung and baseline cognition (b_0_)		
Intervention ^1^	−0.372 (0.099)	**<0.001**
Control ^1^	−0.244 (0.100)	**0.015**
Best model; combined ^2^	−0.302 (0.070)	**<0.001**
Baseline Zung and cognitive change (b_1_)		
Intervention ^1^	−0.142 (0.085)	0.095
Control ^1^	−0.103 (0.087)	0.235
Best model; combined ^2^	−0.140 (0.050)	**0.005**
Zung change and cognitive change (b_3_)		
Intervention ^1^	−0.124 (0.776)	0.873
Control ^1^	−0.404 (0.773)	0.601
Best model; combined ^2^	constrained to 0	N/A
**Executive functioning domain**		
Baseline Zung and baseline cognition (b_0_)		
Intervention ^1^	−0.393 (0.120)	**0.001**
Control ^1^	−0.270 (0.122)	**0.027**
Best model; combined ^3^	−0.360 (0.081)	**<0.001**
Baseline Zung and cognitive change (b_1_)		
Intervention ^1^	−0.096 (0.113)	0.396
Control ^1^	−0.038 (0.109)	0.726
Best model; combined ^3^	constrained to 0	N/A
Zung change and cognitive change (b_3_)		
Intervention ^1^	0.593 (0.926)	0.522
Control ^1^	−1.085 (0.915)	0.235
Best model/intervention ^3^	constrained to 0	N/A
Best model/control ^3^	−1.371 (0.736)	0.063
**Memory domain**		
Baseline Zung and baseline cognition (b_0_)		
Intervention ^1^	−0.218 (0.127)	0.087
Control ^1^	−0.160 (0.120)	0.185
Best model; combined ^4^	−0.177 (0.088)	**0.043**
Baseline Zung and cognitive change (b_1_)		
Intervention ^1^	−0.238 (0.130)	0.068
Control ^1^	−0.166 (0.132)	0.210
Best model; combined ^4^	−0.231 (0.083)	**0.005**
Zung change and cognitive change (b_3_)		
Intervention ^1^	−0.697 (1.108)	0.529
Control ^1^	−0.296 (1.080)	0.784
Best model; combined ^4^	constrained to 0	N/A
**Processing speed domain**		
Baseline Zung and baseline cognition (b_0_)		
Intervention ^1^	−0.648 (0.145)	**<0.001**
Control ^1^	−0.344 (0.147)	**0.019**
Best model; combined ^4^	−0.540 (0.100)	**<0.001**
Baseline Zung and cognitive change (b_1_)		
Intervention ^1^	−0.069 (0.112)	0.535
Control ^1^	−0.120 (0.108)	0.268
Best model; combined ^4^	constrained to 0	N/A
Zung change and cognitive change (b_3_)		
Intervention ^1^	−1.051 (0.984)	0.286
Control ^1^	0.604 (0.851)	0.478
Best model; combined ^4^	constrained to 0	N/A

Note: Path refers to Figure S1 and Table S2 in Supplementary material. Baseline refers to the latent intercept and change refers to the latent slope estimated with parallel growth curves. All models are adjusted for age, sex, education, trial site, antidepressant use, and healthy lifestyle change index. *p*-values < 0.05 are shown in bold. ^1^ Model 2 (see Supplementary Methods in Supplementary material for details), where measurement error covariances are estimated as equal between groups. ^2^ Model 6 (see Supplementary Methods in Supplementary material for details), where all parameters are estimated as equal and the path slope (Zung)-slope (cognition) is constrained to [0]. ^3^ Model 6 (see Supplementary Methods in Supplementary material for details), where the path slope (Zung)-slope (cognition) is constrained to [0] in the intervention group. ^4^ Model 7 (see Supplementary Methods in Supplementary material for details), where all parameters are estimated as equal and the path intercept (Zung)-slope (cognition) is constrained to [0].

**Table 3:** The effect of clinically significant depressive symptoms at baseline on change in cognition (results of the best-fitting model).

	Global Cognition	Executive Functioning	Memory	Processing Speed
	Estimate (95 % CI), *p*-Value			
Baseline Zung and baseline cognition (“Zung”)	−0.153 (−0.226–−0.079), *p* < 0.001	−0.140 (−0.267–−0.013), *p* = 0.031	−0.148 (−0.240–−0.055), *p* = 0.002	−0.148 (−0.257–−0.039), *p* = 0.008
Baseline Zung and change in cognition (“Time × Zung”)	−0.035 (−0.062–−0.008), *p* = 0.010	0.014 (−0.033–0.061), *p* = 0.562	−0.044 (−0.088–−0.0001), *p* = 0.049	−0.033 (−0.069–0.004), *p* = 0.081
Impact on intervention effect (“Group × time × Zung”)	N/A	−0.096 (−0.163–−0.028), *p* = 0.005	N/A	N/A

Note: The best-fitting mixed-effects regression model is based on the lowest accepted Bayesian Information Criterion (BIC) shown in Table S4 in Supplementary material. Analyses are adjusted for age, sex, education, trial site, antidepressant use, and healthy lifestyle change index. Other parameters included in the best-fitting model: “Randomisation group”, “Time”, “Group × time”, and “Group × Zung” (see Table S3 in Supplementary material).

## Data Availability

Data used in this study are not publicly available due to ethical and legal reasons, but the data are available upon request. Those fulfilling the requirements for viewing confidential data as required by the Finnish legislation and the Finnish Institute for Health and Welfare are able to access the data after completion of a material transfer agreement. Requests may be directed to kirjaamo@thl.fi.

## Supplementary Materials

The following supporting information can be downloaded at: https://www.mdpi.com/article/10.3390/jcm11051449/s1, Figure S1: Graphical presentation of the growth curve model, Table S1: Baseline characteristics of the FINGER trial population with baseline Zung data, Supplementary Methods: Modelling and model selection of parallel process latent growth curves, Table S2: Model fit and model comparison for all nested parallel growth curve models of cognition and Zung score, Table S3: The effect of clinically significant depressive symptoms at baseline on change in cognition (results of the best-fitting model), Table S4: Assessment of the best-fitting model regarding the effects of baseline dichotomised Zung score using Bayesian Information Criterion (BIC).
