# Supplementary material for: Associations of Depressive Symptoms and Cognition in the FINGER Trial: A Secondary Analysis of a Randomised Clinical Trial
Source: J Clin Med. 2022 Mar 7;11(5):1449. doi: 10.3390/jcm11051449 (PMC8911355; doi:10.3390/jcm11051449)
Supplement: Supplementary file 1 [file jcm-11-01449-s001.zip › jcm-1506762-supplementary.pdf]

Supplementary material

Supplement 1. Table S1: Baseline characteristics of the FINGER trial population with baseline Zung data

Supplement 2. Supplementary Methods: Modelling and model selection of parallel process latent growth curves

Supplement 3. Figure S1: Graphical presentation of the growth curve model

Supplement 4. Table S2: Model fit and model comparison for all nested parallel growth curve models of cognition and Zung score

Supplement 5. Table S3: The effect of clinically significant depressive symptoms at baseline on change in cognition (results of the best-fitting model)

Supplement 6. Table S4: Assessment of the best-fitting model regarding the effects of baseline dichotomised Zung score using Bayesian Information Criterion (BIC)

Supplement 1. Table S1: Baseline characteristics of the FINGER trial population with baseline Zung data

Baseline variables	Number of persons with available information	Intervention group (n=550)	Control group (n=575)	p-value
Age (years)	1125	69.3 (4.6)	69.2 (4.7)	0.47
Sex: female	1125	239/550 (43%)	263/575 (46%)	0.44
Education (years)	1124	10.1 (3.5)	10.0 (3.4)	0.75
Habitation: co/married	1125	408/550 (74%)	440/575 (77%)	0.36
SBP (mmHg)	1117	140 (17)	140 (16)	0.47
DBP (mmHg)	1117	80 (10)	80 (9)	0.95
Cholesterol (mmol/l)	1120	5.2 (1.0)	5.1 (1.0)	0.29
Glucose (mmol/l)	1122	6.1 (0.8)	6.1 (0.9)	0.90
2h OGTT (mmol/l)	972	7.0 (2.2)	7.0 (2.1)	0.86
Body Mass Index (kg/m^2)	1115	28.2 (4.5)	28.0 (4.9)	0.44
Waist circumference (cm)	1116	98.6 (12.2)	97.8 (12.8)	0.46
APOE ε4 allele carrier	1046	161/513 (31%)	189/533 (35%)	0.16
Physically active	1117	385/546 (71%)	408/571 (71%)	0.73
Smoker	1123	57/548 (10%)	47/575 (8%)	0.20
Alcohol use ≥1/week	1120	252/548 (46%)	263/572 (46%)	1.00
Fish use ≥2/week	1122	287/548 (52%)	289/574 (50%)	0.50
Vegetable use daily	1124	339/549 (62%)	359/575 (62%)	0.81
Hypertension	1118	359/548 (66%)	374/570 (66%)	0.97
Hypercholesterolaemia	1115	356/546 (65%)	393/569 (69%)	0.17
Diabetes	1119	72/548 (13%)	75/571 (13%)	1.00
MI history	1119	30/548 (5%)	30/571 (5%)	0.87
Stroke history	1116	26/546 (5%)	33/570 (6%)	0.44
Depression diagnosis	1114	31/548 (6%)	37/566 (7%)	0.54
Antidepressant use	1125	29/550 (5%)	33/575 (6%)	0.73
Zung depression score	1125	33.9 (7.8)	33.9 (7.2)	0.63
Global cognition	1124	-0.01 (0.56)	0.01 (0.58)	0.47
Executive function	1123	-0.01 (0.66)	0.01 (0.69)	0.58
Memory	1124	-0.01 (0.69)	0.01 (0.65)	0.42
Processing speed	1124	-0.004 (0.77)	0.02 (0.84)	0.44
MMSE score	1122	26.7 (2.0)	26.8 (2.0)	0.61

Values are expressed as mean (SD), and t-test or rank-sum were used to compare differences between the groups for continuous variables; values are expressed as n/N (%) and chi-square test was used for categorised variables. APOE, apolipoprotein E; DBP, diastolic blood pressure; MI, myocardial infarction; MMSE, Mini-Mental State Examination; OGTT, oral glucose tolerance test; SBP, systolic blood pressure

Supplement 2. Supplementary Methods: Modelling and model selection of parallel process latent growth curves

Analyses were performed using structural equation modelling (SEM) with SPSS Amos, exploiting the group option which allows parameters in the same model to be estimated as unequal for two groups (here intervention and control). Analyses were initiated with a theoretical full path model where all parameters were estimated as unequal, as if there were two separate models (Figure S1). The final model was selected constraining parameters as equal between groups one at a time, each model compared with the full model using likelihood ratio test. Time was entered as non-linear for both Zung score and cognition, based on best fit in previous univariate models, by not constraining the factor loading for latent slope (change) of the middle year [0, free, 1]. Factor loadings for latent intercept (baseline level) were constrained to [1,1,1].

Other constraints were tested by comparing nested models against the theoretical full model (Model 0) regarding global cognition and memory domain as follows: Model 1) Constrained based on univariate models (factor loadings, path coefficients, residuals, means, variances, and covariances set equal between groups except for cognition intercept and slope residuals, estimated intercepts, and slopes; additionally Zung score slope-intercept covariance set to 0); Model 2) Measurement error covariances set as equal; Model 3) path intercept (Zung) → intercept (cognition) estimated as equal; Model 4) intercept (Zung) → slope (cognition) estimated as equal; and Model 5) slope (Zung) → slope (cognition) set as equal between groups, i.e. all parameters estimated as equal between groups as if there were only one single group. We additionally constrained the path between latent slopes, i.e. Model 6) slope (Zung) → slope (cognition) constrained to [0], to examine the role of baseline Zung in cognitive change when changes in Zung score were not included in the model; and the path between intercept and slope, i.e. Model 7) intercept (Zung) → slope (cognition) constrained to [0], to examine the association between baseline Zung and baseline cognition when changes in either were not taken into account.

Regarding processing speed domain, constraints were as follows: Model 1) Constrained based on univariate models (factor loadings, path coefficients, residuals, means, variances, and covariances set equal between groups except for cognition intercept and slope residuals, estimated intercepts, and slopes; additionally Zung score slope-intercept covariance set to 0, processing speed residual intercept-slope covariance set to 0, and slope covariance for cognition set equal between groups); Model 2) Measurement error covariances set as equal; Model 3) path intercept (Zung) → intercept (cognition) estimated as equal; Model 4) intercept (Zung) → slope (cognition) estimated as equal; and Model 5) slope (Zung) → slope (cognition) set as equal between groups, i.e. all parameters estimated as equal between groups as if there were only one single group. Like earlier described, in Model 6) slope (Zung) → slope (cognition) was constrained to [0]; and in Model 7) intercept (Zung) → slope (cognition) was constrained to [0].

Regarding executive function domain, constraints were as follows: Model 1) Constrained based on univariate models (factor loadings, path coefficients, residuals, means, variances, and covariances set equal between groups except for cognition intercept and slope residuals, estimated intercepts, and slopes; additionally Zung score slope-intercept covariance set to 0); Model 2) Measurement error covariances set as equal; Model 3) path intercept (Zung) → intercept (cognition) set as equal; and Model 4) path intercept (Zung) → slope (cognition) set as equal between groups. In Model 5) path intercept (Zung) → slope (cognition) was constrained to [0]; in Model 6) slope (Zung) → slope (cognition) for intervention group only was constrained to [0]; and in Model 7) slope (Zung) → slope (cognition) for control group was constrained to [0].

Adequate model fit was considered with a chi-square test p-value > 0.05 (based on χ^2 value and degrees of freedom, DF); mean square residual (RMSEA) < 0.05, and comparative fit index (CFI) > 0.98. Also, Browne-Cudeck information criterion (BCC) was used to select the best-fitting model (lower value represents better relative fit to data). Model 6 was the best-fitting model and thus considered the final model regarding global cognition and memory domain, as well as for executive function domain with different model constraints. Model 7 was the best-fitting model regarding processing speed domain. Model fits are reported in Table S1.

Supplement 3. Figure S1: Graphical presentation of the growth curve model

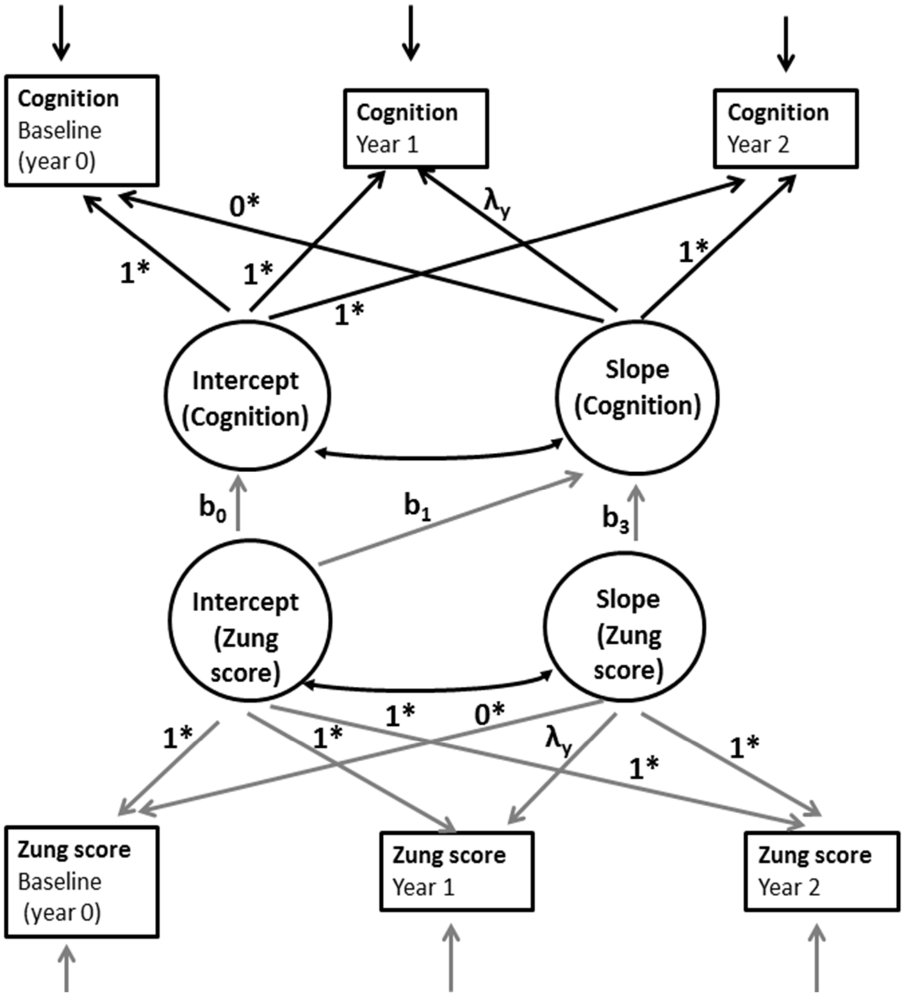

Figure S1: Graphical presentation of the process latent growth curve model

b_0) refers to the path examining the cross-sectional associations of baseline (intercept) Zung score with baseline (intercept) cognition (Baseline Zung → baseline cognition in Table 2); b_1) refers to the path examining the prospective association of baseline (intercept) Zung score with change (slope) in cognition (Baseline Zung → cognitive change in Table 2); and b_3) refers to the parallel associations of changes in Zung score with change in cognition (Zung change → cognitive change in Table 2).

Supplement 4. Table S2: Model fit and model comparison for all nested parallel growth curve models of cognition and Zung score

Nested models[a]	χ^{2b}	DF	BCC	RMSEA	CFI	p-value (LR test)[c]
**Global cognition**						
0) Theoretical full path model	11.610	12	611.845	0	1	
1) Constrained based on univariate models[d]	21.667	24	597.236	0	1	0.611
2) Measurement error covariances set equal	22.742	27	592.144	0	1	0.783
3) Intercept -> intercept equal	23.608	28	590.954	0	1	0.352
4) Intercept -> slope equal	23.822	29	589.113	0	1	0.644
5) Slope -> slope equal	23.905	30	587.140	0	1	0.774
6) Slope -> slope constrained to [0]	23.996	31	585.175	0	1	0.763
7) Intercept -> slope constrained to [0]	31.829	32	590.953	0	1	0.005
**Executive function domain**						
0) Theoretical full path model	16.689	12	616.924	0.000	1	
1) Constrained based on univariate models[d]	28.216	24	603.784	0.012	0.999	0.484
2) Measurement error covariances set equal	30.628	27	600.029	0.010	0.999	0.491
3) Intercept -> intercept equal	31.172	28	598.518	0.009	0.999	0.461
4) Intercept -> slope equal	31.619	29	596.910	0.008	1	0.504
5) Intercept -> slope constrained to [0]	32.239	30	595.474	0.008	1	0.431
6) Slope -> slope constrained to [0] in intervention	32.305	31	593.484	0.006	1	0.797
7) Slope -> slope constrained to [0] in control	37.716	32	596.839	0.012	0.999	0.020
**Memory domain**						
0) Theoretical full path model	7.484	12	606.229	0	1	
1) Constrained based on univariate models[d]	21.979	24	597.548	0	1	0.270
2) Measurement error covariances set equal	23.715	27	593.116	0	1	0.629
3) Intercept -> intercept equal	23.831	28	591.177	0	1	0.733
4) Intercept -> slope equal	24.100	29	589.390	0	1	0.604
5) Slope -> slope equal	24.454	30	587.688	0	1	0.522
6) Slope -> slope constrained to [0]	24.677	31	585.856	0	1	0.637
7) Intercept -> slope constrained to [0]	32.366	32	591.490	0.003	1	0.006
**Processing speed domain**						
0) Theoretical full path model	17.620	12	617.855	0.019	0.999	
1) Constrained based on univariate models[e]	31.326	27	600.727	0.011	0.999	0.547
2) Measurement error covariances set equal	33.341	30	596.576	0.009	1	0.569
3) Intercept -> intercept equal	35.570	31	596.749	0.011	0.999	0.135
4) Intercept -> slope equal	35.570	32	594.694	0.009	0.999	0.990
5) Slope -> slope equal	38.521	33	595.589	0.012	0.999	0.086
6) Slope -> slope constrained to [0]	38.610	34	593.622	0.010	0.999	0.766
7) Intercept -> slope constrained to [0]	40.687	35	593.643	0.011	0.999	0.150

Note: χ^2, chi-square test; DF, degrees of freedom; BCC, Browne-Cudeck information criterion; RMSEA, root mean square error of approximation; CFI, comparative fit index

[a] All models are adjusted for age, sex, education, healthy lifestyle change index, trial site, and antidepressant use.

[b] All chi-square test p-values (based on χ^2 value and DF) nonsignificant.

[c] All nested models tested against full model (model 0) with likelihood ratio test.

[d] Model constrained based on univariates includes factor loadings, path coefficients, residuals, means, variances, and covariances set equal between groups, except for cognition intercept and slope residuals, estimated intercepts, and slopes; additionally, Zung score slope-intercept covariance set to 0.

[e] Model constrained based on univariates includes factor loadings, path coefficients, residuals, means, variances, and covariances set equal between groups, except for cognition intercept and slope residuals, estimated intercepts, and slopes; additionally, Zung score slope-intercept covariance set to 0, processing speed residual intercept-slope covariance set to 0, and slope covariance for cognition set equal between groups.

Supplement 5. Table S3: The effect of clinically significant depressive symptoms at baseline on change in cognition (results of the best-fitting model)

Model terms	Global cognition	Executive functioning	Memory	Processing speed
	Estimate (95 % CI), p-value			
**Randomisation group**	-0.018 (-0.076-0.039), p=0.533	-0.033 (-0.114-0.047), p=0.414	-0.018 (-0.088-0.053), p=0.626	0.006 (-0.077-0.090), p=0.878
**Zung**	-0.153 (-0.226- -0.079), p<0.001	-0.140 (-0.267- -0.013), p=0.031	-0.148 (-0.240- -0.055), p=0.002	-0.148 (-0.257- -0.039), p=0.008
**Time**	0.104 (0.092-0.116), p<0.001	0.028 (0.007-0.049), p=0.009	0.178 (0.158-0.198), p<0.001	0.050 (0.034-0.067), p<0.001
**Group x time**	N/A	0.043 (0.013-073), p=0.005	N/A	N/A
**Group x Zung**	N/A	0.010 (-0.170-0.191), p=0.912	N/A	N/A
**Time x Zung**	-0.035 (-0.062- -0.008), p=0.010	0.014 (-0.033-0.061), p=0.562	-0.044 (-0.088- -0.0001), p=0.049	-0.033 (-0.069-0.004), p=0.081
**Group x time x Zung**	N/A	-0.096 (-0.163- -0.028), p=0.005	N/A	N/A

Note: The best-fitting mixed-effects regression model is based on the lowest accepted Bayesian Information Criterion (BIC) shown in Table S3 in Supplementary material. Analyses are adjusted for age, sex, education, trial site, antidepressant use, and healthy lifestyle change index.

Supplement 6. Table S4: Assessment of the best-fitting model regarding the effects of baseline dichotomised Zung score using Bayesian Information Criterion (BIC)

Full model	Global cognition	Executive function	Memory	Processing speed
	BIC[a]			
- Randomisation group<br>- Zung<br>- Time<br>- Group x time<br>- Group x Zung<br>- Time x Zung<br>- Group x time x Zung	2024.699	**3536.958**	4423.284	4226.182
**Alternative models**	**BIC[a] (p-value for comparison with the full model)[b]**			
**Model 1**<br>- Randomisation group<br>- Zung<br>- Time<br>- Group x time<br>- Group x Zung<br>- Time x Zung	2018.392<br>(0.1958)	3536.704<br>(0.0055)	4415.323<br>(0.8894)	4218.279<br>(0.7818)
**Model 2**<br>- Randomisation group<br>- Zung<br>- Time<br>- Group x time<br>- Time x Zung	2011.725<br>(0.2246)	3529.263<br>(0.0161)	4409.353<br>(0.3624)	4211.085<br>(0.6499)
**Model 3**<br>- Randomisation group<br>- Zung<br>- Time<br>- Group x time<br>- Group x Zung	2016.993<br>(0.0161)	3532.237<br>(0.0036)	4411.2<br>(0.1439)	4213.349<br>(0.2096)
**Model 4**<br>- Randomisation group<br>- Zung<br>- Time<br>- Time x Zung	**2006.335<br>(0.1341)**	3524.269<br>(0.0105)	**4401.686<br>(0.5043)**	**4206.465<br>(0.2386)**

Note: The best-fitting mixed-effects regression model (lowest BIC with nonsignificant LR test, bold font) was determined by performing likelihood ratio tests and comparing alternative models with the full model excluding non-significant interaction terms. All analyses are adjusted for age, sex, education, trial site, use of antidepressants, and healthy lifestyle change index.

[a] BIC = -2*log-likelihood + log(number of observations)*(number of estimated parameters)

[b] p-value from likelihood ratio test comparing each alternative model with the full model
